# Changes in executive function in the Canadian longitudinal study on aging over 3-years: A focus on social determinants of health

**DOI:** 10.3389/fpsyg.2023.1060178

**Published:** 2023-01-27

**Authors:** Arne Stinchcombe, Nicole G. Hammond, Shawna Hopper

**Affiliations:** ^1^School of Psychology, University of Ottawa, Ottawa, ON, Canada; ^2^Bruyère Research Institute, Ottawa, ON, Canada; ^3^School of Epidemiology and Public Health, University of Ottawa, Ottawa, ON, Canada; ^4^Department of Gerontology, Simon Fraser University, Vancouver, BC, Canada

**Keywords:** executive function, aging, social determinants of health, cognition, social identity, CLSA

## Abstract

Maintaining executive functions, including planning, inhibition, and decision-making skills, is important for autonomy and activities of daily living. There is a growing body of evidence linking social determinants and cognitive aging, but less is known about the potential role of social determinants in changes in executive functioning over time. Using data from the Canadian Longitudinal Study on Aging (CLSA), a large cohort of mid-aged and older adults, we examined changes in executive function over a 3-year period. Specifically, we focused on the role of social determinants (i.e., social positioning, social support, education) in explaining these changes. Executive function was measured at baseline and follow-up 3 years later using the Mental Alteration Test (MAT). We computed a reliable change index (RCI) and used a multiple linear regression model to examine the associations between known correlates and change in executive function over the 3-year period (*n* = 29,344). Older age, higher household income, and greater educational attainment predicted declines in executive function. Health factors (e.g., depression symptoms, physical activity levels) and many social determinants (sexual orientation, gender identity, race, and perceived social standing) were not associated with change in executive function. These results suggest that social determinants of health may be related to initial differences in cognitive functioning (i.e., cross-sectional differences) rather than more rapid cognitive aging.

## Introduction

As life expectancy increases globally, the number of people living with cognitive impairments, such as Alzheimer’s disease (AD), is increasing (Prince et al., 2016). Cognition is essential for preforming everyday tasks, thus maintaining cognitive abilities into older age is important for autonomy and independence in daily living (Marshall et al., 2011). For most, slight age-related declines in cognitive abilities (i.e., executive function, episodic memory) begin during mid-life and are considered a normal part of aging (Salthouse, 2009). However, substantial changes in cognition may be an indication of the onset of a cognitive impairments or dementia.

Executive function refers to the higher-order cognitive skills necessary for reasoning and problem solving. Executive functioning includes an individual’s inhibition, mental flexibility, and the ability to plan (Chen et al., 1998; Carlson et al., 2013). These skills are particularly important in older age as they are necessary for numerous activities that older adults preform on a daily basis (Overdorp et al., 2016). Executive functions have been linked to older adults’ ability to compensate for age-associated changes in other cognitive functions, such as memory (Bouazzaoui et al., 2014). Research has found that low executive function is associated with impairment of instrumental activities of daily living (IADL) and are predictive of future IADL decline (Overdorp et al., 2016). For example, individuals experiencing declines in executive functioning may have difficulties with tasks such as shopping, laundry, financial management, and transportation (Jefferson et al., 2006). In addition to being associated with activities of daily living (ADLs) and IADLs, executive dysfunction is associated with an increased risk of mortality (Johnson et al., 2007). Significant deficits in executive function impact not only the quality of life of the individual, but also their loved ones, as it adds substantial burden to caregivers (Marshall et al., 2011). Further, declines in executive function have been associated with an increased risk of cognitive impairment, and may be one of the earliest indicators of AD, preceding declines in memory (Carlson et al., 2009). A study by Carlson et al. (2009) found that declines in executive function preceded memory declines by 3 years, and increased risk of global cognitive impairment. This means that measures of executive function may be useful in identifying individuals with AD before they meet clinical criteria (Albert et al., 2001), and help implement early interventions to prevent future cognitive decline (Carlson et al., 2009).

Recent literature has focused on identifying modifiable risk factors of dementia, in order to prevent or delay cognitive decline (Livingston et al., 2020). These risk factors include hypertension, alcohol intake, obesity, smoking, depression, social isolation, and more (Livingston et al., 2020). Although ample research has focused on correlates of overall cognitive decline (Plassman et al., 2010; Baumgart et al., 2015), less research has focused on specific risk and protective factors for executive function. Recently, a population-based cross-sectional study by Stinchcombe and Hammond (2021) identified correlates of executive function, similar to those identified by Livingston et al. (2020). For example, smoking, hypertension, sensory issues, and less social support were associated with lower levels of executive function (Stinchcombe and Hammond, 2021). Associations between cognitive, physical, and social engagement and executive function have been found in both cross-sectional, and longitudinal analyses. Individuals with increased social engagement performed better on executive functioning tasks, and showed stability or improvement in executive functioning over time (de Frias and Dixon, 2014). Similarly, other research on older adults has found that participating in physical activity (i.e., aerobic training) can help retain executive functioning over time (Daly et al., 2015), and is associated with improvements in executive function (Erickson et al., 2019).

Evidence suggests that the individual differences in later-life cognition may be due to psychological and social factors increasing one’s cognitive reserve. The cognitive reserve theory hypothesizes that individuals build up their cognitive abilities over the lifespan, which allows them to better tolerate age-related brain changes and delay decline in cognitive function (Stern, 2012). Lifetime exposures that contribute to one’s cognitive reserve include educational attainment, occupation, physical activity, leisure participation, and social engagement (Stern et al., 2020). These proxy measures of cognitive reserve (i.e., education, occupation, engagement in cognitively stimulating activities) have been positively associated with executive function in later life (Opdebeeck et al., 2016). Further, cognitive reserve has been found to moderate the negative effect of aging on executive function (Giogkaraki et al., 2013), and slow decline in executive function (Reed et al., 2010).

In addition to health and cognitive reserve factors, cognition among older adults is influenced by the structural and social determinants of health (SSDoH). SSDoH are understood as the social and physical environmental conditions where people are born, live, learn, work, play and worship, which have a major impact on people’s health, well-being, and quality of life (Healthy People 2030, 2022). Responding to a growing recognition of the importance of SSDoH in regards to Alzheimer’s risk, likelihood of diagnosis, and prognosis, Stites et al. (2022) proposed a framework outlining SSDoHs that may help better understand the determinants and mechanisms of Alzheimer’s disease and related dementias. Their framework identifies seven core domains for SSDoH in relation to dementia: social stressors and perceived stress, social support, education and health literacy, occupation, social positioning, social/built environment/neighborhood, and social identity.

Relatedly, Forrester et al. (2019) proposed a biopsychosocial framework of minority aging which suggests that psychosocial factors throughout the lifespan play a key role in the disparity in cognitive ability in later life. Their model depicts the relationship between psychosocial factors, behavior factors, and allostatic load; noting that the accumulated impact of these conditions on minoritized individuals leads to increased cognitive impairment (Forrester et al., 2019). This framework aligns with that of Stites et al. (2022), which acknowledges the roles of social positioning (i.e., income and social status) and social identity (i.e., gender, race, and sexual orientation) in cognition. For example, research has found cross-sectional differences in executive functioning among members of racialized communities (Chen et al., 2021; Stinchcombe and Hammond, 2021), that may be explained by the social inequities faced by non-White persons (Rea-Sandin et al., 2021). Furthermore, although Black individuals have been found to have lower baseline executive functioning, longitudinal research has found that decline in executive functioning is slower among Black individuals compared to White individuals (Weuve et al., 2018). While individuals with minoritized identities may be at an increased risk of cognitive decline (Correro and Nielson, 2020), they are also underrepresented in research (Bonevski et al., 2014). To our knowledge, no research has yet to examine the associations between other minority identities (i.e., sexual orientation, gender identity) and changes in executive function over time.

Given the importance of executive function for independent living, we sought to examine the role of biopsychosocial factors in explaining changes in executive function over a 3-year period in a national population-based sample. Specifically, we were interested in the extent that social identities (i.e., race, gender identity, and sexual orientation) more likely to experience minority stress were associated with changes executive function over time. It was anticipated that individuals with minoritized identities would have a greater decline in executive function between time one and time two. Understanding changes in executive function over 3-years in a sample of mid-life and older adults who were cognitively healthy at baseline can shed light into cognitive aging trajectories and potential risk for cognitive impairment.

## Materials and Methods

### Dataset

Data reported here is from two time points (baseline and first follow-up) of the Canadian Longitudinal Study on Aging (CLSA), a national, population based, prospective cohort study of mid-life and older adults. The CLSA aims to collect data every 3 years for at least 20 years, or until participant death. Recruitment started in 2010, with baseline data collection completed in 2015, and first follow-up data collection completed in 2018 (Raina et al., 2019). English and French speaking participants were recruited through the Canadian Community Health Survey (CCHS) on Healthy Aging, provincial health registration databases, and random-digit dialing (Raina et al., 2009). Individuals with a cognitive impairment were excluded at baseline, along with institutionalized populations, individuals living in Canadian territories or certain remote areas, those living on First Nations reserves or First Nations settlements, and full-time members of the Canadian Armed Forces (Raina et al., 2008).

The CLSA is comprised of a Tracking cohort (*n* = 21,241) and a Comprehensive Cohort (*n* = 30,097). The tracking cohort participates in computer-assisted telephone interviews (CATI) whereas the comprehensive cohort participates in CATI interviews, in-person home interviews, as well as additional data collection (i.e., additional tests, physical measurements, biospecimen collection) at one of 11 data collection sites (DCS) across Canada (Raina et al., 2019). Participants in the comprehensive cohort must live within 25–50 km of a DCS. Additionally, to maintain participant retention, all participants were administered a 30-min telephone-based interview between baseline and follow-up one: The Maintaining Contact Questionnaire (MCQ). All participants gave written informed consent. Ethical review of the CLSA protocol was conducted by the Ethical, Legal, and Social Issues Committee, falling under the jurisdiction of the Canadian Institutes of Health Research (CIHR), and research ethics board approval was then acquired from each research site. The University of Ottawa REB approved the analyses presented here.

### Executive function (outcome)

The Mental Alternation Test (MAT; Jones et al., 1993; Teng, 1995) was administered to both the CLSA tracking (Tuokko et al., 2017) and comprehensive (Tuokko et al., 2020) cohorts. The MAT is a brief instrument that estimates executive functions; it consists of a cognitive switching task that measures mental flexibility and processing speed (Teng, 1995; Tuokko et al., 2017). Participants were asked to alternate between numbers (1–26) and alphabetical letters in sequence, and as quickly as possible, in 30 s (i.e., 1-A-2-B-3-C, etc.; Tuokko et al., 2017). MAT scores reflect the total number of correct mental alternations, with a possible maximum of 52 (Tuokko et al., 2020). The MAT has good retest reliability (*r* = 0.80–0.90; Jones et al., 1993; Teng, 1995). Further, MAT scores strongly correlate with and predict Mini-Mental State Examination (MMSE; Folstein et al., 1975) scores in different populations (Jones et al., 1993; Billick et al., 2001), an established measure for identifying cognitive status.

Given the potential for practice effects of cognitive measures administered repeatedly over time, we computed 3-year reliable change in executive functioning using a Reliable Change Index (RCI; Jacobson and Traux, 1991). The RCI calculation accounted for practice effects (Chelune et al., 1993) and variability in MAT scores at baseline and first follow-up (Iverson, 2001). Duff (2012) presents a detailed overview of the calculations. The RCI was calculated as follows:


$$\mathrm{RCI}=\frac{\mathrm{simple discrepancy score}-\mathrm{mean practice effect}}{SED_{Iverson}}$$

or,


$$\mathrm{RCI}=\frac{\left(T2-T1\right)-\left(M2-M1\right)}{\sqrt{SEM1+SEM2}}$$

Higher (positive) scores reflect more reliable improvements in executive ability across time, whereas lower (negative) scores reflect more reliable deteriorations.

### Predictors

#### Demographic characteristics

Participants were asked to report their age in years (range: 45–85), as well as their sex at birth (male/female). Marital status was recorded and categorized as single/never married, married/common law, and widowed, divorced, or separated.

#### Health measures

Depression symptoms were measured using the Center for Epidemiologic Studies Short Depression Scale (CESD-10; Andresen et al., 1994). The CESD-10 includes 10 questions regarding frequency of depression symptoms in the past week (Andresen et al., 1994). An overall score (range: 0–30) is determined by summing the response values, with a higher score indicating more depressive symptoms. Participants were asked if a doctor has ever told them that they have heart disease (yes/no), as well as high blood pressure or hypertension (yes/no). Body Mass Index (BMI; kg/m^2^) was calculated using participants weight and height. Vision and hearing were both self-reported with each measure having five possible responses: excellent, very good, good, fair, or poor. Responses of “poor” and “fair” were considered to represent low vision/hearing, whereas the other response options were considered to indicate the absence of low vision/hearing. Based on their smoking status, participants were classified as never smokers (smoked <100 cigarettes in their lifetime), former smokers (smoked ≥100 cigarettes in their lifetime but have not smoked in the past month), or current smokers (smoked ≥100 cigarettes in their lifetime and smoked in the past month). Type of drinker was classified based on the frequency of drinking alcohol over the past 12-months. Participants were categorized as non-drinkers (no alcohol consumption in the past 12-months), occasional drinkers (<once a month), and regular drinkers (≥once a month). The Physical Activity Scale for the Elderly (PASE; Washburn et al., 1993) was used during the MCQ to measure participants’ physical activity over the past 7 days, including activities such as walking, housework, yard work, and caring for others. An overall PASE score was calculated based on the frequency and intensity of activities, with scores ranging from 0 to 629.57 (higher scores indicate greater physical activity). For the purpose of this study, physical activity was categorized using quartiles, with the top quartile (Carlson et al., 2013) representing the most physically active participants, and the lowest quartile (Prince et al., 2016) representing the least physically active participants. Self-rated general health was recorded by asking participants if in general they would say their health was, excellent, very good, good, fair, or poor.

#### Education

Participants were asked to report their highest level of education completed. Responses were categorized as: <secondary school education, secondary school/some post-secondary school education, and post-secondary school education.

#### Occupation

Current retirement status was self-reported and categorized as: not-retired, partly retired, and completely retired.

#### Social support

A continuous measure of social support was captured using the Medical Outcomes Study Social Support Survey (MOS-SSS; Sherbourne and Stewart, 1991). The MOS-SSS is a 19-item scale generating an overall score between 0 and 100, with higher scores indicating greater overall perceived support. Participants also indicated if they have a hobby or pastime (yes/no).

#### Social positioning

Income was captured by asking participants to report their household income in the past 12 months. For the purposes of this study, we categorized income as <$50,000, $50,000–99,999, $100,000–149,000, and ≥$150,000. In the MCQ, participants reported their perceived social standing in their community using the MacArthur Scale of Subjective Social Status (Adler et al., 2000). Participants were asked to think of a ladder with 10 rungs, with the bottom rung (Prince et al., 2016) representing people with the lowest social standing in their community, and the top rung (Carlson et al., 2009) representing people with the highest social standing in their community. They were then asked to place themselves on this ladder (range: 1–10).

#### Social identity

To capture sexual orientation, at first follow-up participants were asked if they consider themselves to be: heterosexual, homosexual (lesbian/gay), bisexual, or other (does not identify with any of the responses). At first follow-up participants were also asked their current gender identity. For the purpose of this study participants were classified as cis-gender (male/female) and transgender/gender diverse (transman, transwoman, genderqueer). Participants who responded as “other” then provided more information through an open-ended response (*n* = 15). Based on this information, members of the research team interpreted the responses and recategorized them as cis-gender or transgender/gender diverse. Race/ethnicity was self-reported and categorized as White, Black, and other non-White.

#### Cohort membership

As previously mentioned, the tracking and comprehensive cohorts undergo different data collection methods. To account for potential differences between the cohorts, we included an indicator of cohort membership (tracking/comprehensive).

### Statistical analysis

Sample characteristics are described using percentages (%) and means (*M*) and standard deviations (*SD*). The primary statistical analysis consisted of a multiple linear regression treating the RCI for executive function as the outcome. Participants who reported cognitive impairment at baseline and those who did not participate in the Maintaining Contact Questionnaire were excluded. Accounting for missing data and attrition resulted in the analytic sample of *n* = 29,344. Participants who were lost to follow-up (*n* = 3,520) were more likely to be older (mean age of 66.35 years compared to 62.64 years; *p* < 0.001) and part of the tracking cohort (*p* < 0.001). All predictors were simultaneously entered into the model. Additionally, we conducted a sensitivity analysis using a simple executive function discrepancy score (Mean_Time2_ – Mean_Time1_) as the outcome instead of the RCI. We did not employ CLSA-derived sampling weights as unweighted, and weighted regression-based parameter estimates for the MAT have been found not to differ in the CLSA (O’Connell et al., 2019). Results, **where** *p* < 0.05, were considered statistically significant. We used Stata version 15.1 (College Station, TX, United States: StataCorp LLC) for the analyses.

## Results

### Sample characteristics

Characteristics of participants in the analytic sample are presented in Table 1. The mean age of participants was 61.6 years old at baseline. There was representation from female (49.7%) and male (50.3%) participants. Most participants (77.7%) reported having completed post-secondary education. In terms of retirement status, 59.4% of participants were retired or partially retired. In terms of social identity, 1.8% of the sample was gay or lesbian while 0.6% of the sample was bisexual. The majority of the sample was White (95.9%).

**Table 1:** Baseline characteristics of the study population (*n* = 29,334).

Variables	*M* (*SD*)/*n* (%)		
***Demographic variables***		***Education***	
**Age**	61.6 (9.9)	<Secondary school grad	1,424 (4.9%)
**Sex at birth**		Secondary school grad/some post-secondary	5,128 (17.5%)
Male	14,768 (50.3%)	Post-secondary grad	22,792 (77.7%)
Female	14,576 (49.7%)	***Occupation***	
**Marital status**		**Retirement status**	
Single	2,274 (7.8%)	Not retired	14,153 (48.2%)
Married/Common-law	21,502 (73.3%)	Partly retired	3,291 (11.2%)
Widowed/Divorced/Separated	5,568 (19.0%)	Completely retired	11,900 (40.6%)
***Health variables***		***Social support***	
**Depressive symptoms**	5.0 (4.4)	**Social support**	82.9 (16.5)
**Heart disease**		**Hobby**	
No	26,562 (90.5%)	No	2,094 (7.1%)
Yes	2,782 (9.5%)	Yes	27,250 (92.9%)
**Hypertension**		***Social positioning***	
No	19.130 (65.2%)	**Income**	
Yes	10,214 (34.8%)	<$50,000	7,515 (25.6%)
**Body mass index (BMI)**	27.8 (5.3)	$50,000–$99,999	10,760 (36.7%)
**Low vision**		$100,000–$149,999	5,975 (20.4%)
No	27,375 (93.3%)	$150,000+	5,094 (17.4%)
Yes	1,969 (6.7%)	**Perceived social standing**	6.1 (1.9)
**Low hearing**		***Social identity***	
No	26,173 (89.2%)	**Sexual orientation**	
Yes	3,171 (10.8%)	Heterosexual	28,581 (97.4)
**Smoking status**		Lesbian/Gay	530 (1.8%)
Never	13,830 (47.1%)	Bisexual	173 (0.6%)
Former	13,141 (44.8%)	Other	60 (0.2%)
Current	2,373 (8.1%)	**Gender identity**	
**Type of drinker**		Cis gender	29,326 (99.9%)
Non-drinker	2,996 (10.2%)	Trans gender/Gender diverse	18 (0.1%)
Occasional drinker	3,757 (12.8%)	**Race**	
Regular drinker	22,591 (77.0%)	White	28,149 (95.9%)
**Physical activity**		Other non-white	1,046 (3.6%)
Quartile 1	6,308 (21.5%)	Black	149 (0.5%)
Quartile 2	7,284 (24.8)	***Cohort membership***	
Quartile 3	7,627 (26.0%)	Tracking	10,601 (36.1%)
Quartile 4	8,125 (27.7%)	Comprehensive	18,743 (63.9%)
**Self-rated general health**			
Poor	357 (1.2%)		
Fair	1,988 (6.8%)		
Good	8,309 (28.3%)		
Very good	12,438 (42.4%)		
Excellent	6,252 (21.3%)		

### Changes in executive function

Table 2 shows the results of the multiple regression analysis. The results indicated that older age (*B* = −0.005, *p* < 0.001) was associated with worsening executive function. In comparison to the lowest income category, participants who reported an annual household income of $100,000–$149,999 showed decreases in executive function over 3 years (*B* = −0.048, *p* = 0.020). There was no association with marital status.

**Table 2:** Results of a multiple linear regression with reliable change index and simple discrepancy score for executive function as the outcome (*n* = 29,334).

Characteristics	Reliable change index				Simple discrepancy score			
	*B*	*p*	CI (lower)	CI (upper)	*B*	*p*	CI (lower)	CI (upper)
*Demographic variables*								
Age^a^	−0.005***	<0.001	−0.007	−0.003	−0.032***	<0.001	−0044	−0.020
Sex at birth								
Male	Referent	–	–	–	Referent	–	–	–
Female	0.005	0.708	−0.020	0.030	0.032	0.708	−0.138	0.203
Marital status								
Single	Referent	–	–	–	Referent	–	–	–
Married/common-law	0.003	0.911	−0.045	0.051	0.019	0.911	−0.308	0.345
Widowed/divorced/separated	−0.014	0.580	−0.062	0.020	−0.098	0.580	−0.445	0.249
*Health variables*								
Depression symptoms^a^	0.000	0.907	−0.003	0.003	0.001	0.907	−0.019	0.021
Heart disease								
No	Referent	–	–	–	Referent	–	–	–
Yes	−0.021	0.321	−0.062	0.200	−0.143	0.321	−0.425	0.139
Hypertension								
No	Referent	–	–	–	Referent	–	–	–
Yes	−0.002	0.888	−0.029	0.025	−0.013	0.888	−0.195	0.169
BMI^a^	0.002	0.172	−0.001	0.004	0.011	0.172	−0.005	0.027
Low vision								
No	Referent	–	–	–	Referent	–	–	–
Yes	0.006	0.800	−0.041	0.053	0.042	0.800	−0.281	0.364
Low hearing								
No	Referent	–	–	–	Referent	–	–	–
Yes	0.012	0.525	−0.026	0.051	0.085	0.525	−0.176	0.346
Smoking status								
Never	Referent	–	–	–	Referent	–	–	–
Former	−0.003	0.829	−0.027	0.022	−0.019	0.829	−0.188	0.150
Current	0.038	0.096	−0.007	0.084	0.262	0.096	−0.047	0.571
Type of drinker								
Non-drinker	Referent	–	–	–	Referent	–	–	–
Occasional drinker	−0.009	0.725	−0.058	0.040	−0.060	0.725	−0.393	0.273
Regular drinker	−0.012	0.556	−0.051	0.028	−0.081	0.556	−0.350	0.188
Physical activity								
Quartile 1	Referent	–	–	–	Referent	–	–	–
Quartile 2	−0.011	0.549	−0.045	0.024	−0.072	0.549	−0.308	0.164
Quartile 3	−0.014	0.430	−0.049	0.021	−0.096	0.430	−0.333	0.142
Quartile 4	0.004	0.839	−0.033	0.040	0.026	0.839	−0.222	0.274
General health								
Poor	Referent	–	–	–	Referent	–	–	–
Fair	−0.003	0.955	−0.118	0.111	−0.022	0.955	−0.805	0.760
Good	0.047	0.400	−0.062	0.156	0.321	0.400	−0.426	1.068
Very good	0.030	0.596	−0.080	0.140	0.204	0.596	−0.549	0.957
Excellent	0.028	0.631	−0.085	0.141	0.190	0.631	−0.583	0.962
*Education*								
<Secondary school	Referent	–	–	–	Referent	–	–	–
Secondary school/some post-secondary	−0.097**	0.002	−0.157	−0.037	−0.661**	0.002	−1.072	−0.250
Post-secondary	−0.105***	<0.001	−0.161	−0.048	−0.715***	<0.001	−1.101	−0.329
*Occupation*								
Retirement status								
Not retired	Referent	–	–	–	Referent	–	–	–
Partly retired	0.034	0.113	−0.008	0.076	0.231	0.113	−0.055	0.517
Completely retired	0.003	0.865	−0.032	0.038	0.021	0.865	−0.221	0.262
*Social support*								
Social support^a^	0.000	0.869	−0.001	0.001	0.000	0.869	−0.006	0.005
Hobby								
No	Referent	–	–	–	Referent	–	–	–
Yes	0.006	0.811	−0.040	0.051	0.038	0.811	−0.274	0.350
*Social positioning*								
Income								
<$50K	Referent	–	–	–	Referent	–	–	–
$50–99,999	−0.010	0.537	−0.043	0.022	−0.070	0.537	−0.293	0.152
$100–149,999	−0.048*	0.020	−0.088	−0.008	−0.325*	0.020	−0.600	−0.051
≥$150K	−0.038	0.094	−0.082	0.006	−0.257	0.094	−0.558	0.044
Perceived social standing^a^	0.002	0.464	−0.004	0.009	0.017	0.464	−0.028	0.061
*Social identity*								
Sexual orientation								
Heterosexual	Referent	–	–	–	Referent	–	–	–
Lesbian/Gay	−0.028	0.531	−0.117	0.060	−0.192	0.531	−0.795	0.410
Bisexual	−0.001	0.986	−0.153	0.150	−0.009	0.986	−1.043	1.025
Other	−0.052	0.690	−0.310	0.205	−0.357	0.690	−2.113	1.398
Gender identity								
Cis-gender	Referent	–	–	–	Referent	–	–	–
Transgender/gender diverse	0.065	0.785	−0.403	0.534	0.446	0.785	−2.753	3.645
Race								
White	Referent	–	–	–	Referent	–	–	–
Other non-white	0.048	0.137	−0.015	0.111	0.326	0.137	−0.104	0.757
Black	0.137	0.163	−0.280	0.047	−0.795	0.163	−1.911	0.321
*Cohort membership*								
Tracking cohort	Referent	–	–	–	Referent	–	–	–
Comprehensive cohort	0.099***	<0.001	0.074	0.123	0.673***	<0.001	0.505	0.841

a = continuous predictor, *p<0.05, **p<0.01, ***p<0.001.

There were no associations between health variables and changes in executive function over the study period. Specifically, there were no statistically significant associations between executive function and depression symptoms, sensory function, and all health behaviors (smoking, drinking, physical activity).

Greater education was associated with worsening executive function. Specifically, compared to individuals with less than a secondary school diploma, participants who had completed secondary (*B* = −0.097, *p* = 0.002), and post-secondary (*B* = −0.105, *p* = <0.001) had statistically significant decreases in executive function. Neither occupation nor having a hobby predicted changes in executive function. None of the social identities included in the model predicted changes in executive function. There was no statistical association between perceived social standing and changes in executive function (*B* = 0.002, *p* = 0.454).

### Sensitivity analysis

We re-ran the multivariable regression model using a simple executive function discrepancy score (Mean_Time2_ – Mean_Time1_) as the outcome instead of the RCI. Results of the supplementary model are presented in Table 2. While there were some differences in the magnitude of the estimates between the primary and supplementary models, there were no differences in which findings were statistically significant or not.

## Discussion

Given the importance of executive function for independent living, we sought to examine changes in executive function over a 3-year period, focusing on the role of social determinants of health. Based on previous work identifying cross-sectional differences in executive function among minoritized older adults, it was hypothesized that minoritized social identities likely to experience minority stress would demonstrate worsening executive function over the 3-year period, in comparison to their majority peers.

Previous cross-sectional research has established that older minoritized individuals tend to have lower baseline cognitive performance compared to majority peers (Chen et al., 2021; Stinchcombe and Hammond, 2021). Contrary to our hypothesis, the results from our analysis show that individuals more likely to encounter minority stress based on social identities did not show significant changes in executive function over a 3-year period.

These results contribute to a growing body of evidence pointing to older members of minoritized communities exhibiting initial differences in cognitive functioning (i.e., cross-sectional differences) but not necessarily experiencing more rapid cognitive aging. Research on racial differences in cognitive aging by Weuve et al. (2018) found that on average Black participants performed worse on cognitive tests (global cognition, episodic memory, and executive function) compared to White participants. Longitudinally however, the rate of change over a 5-year interval for measures of global cognition and episodic memory did not differ between racial groups. The authors suggest that despite the difference in the rate of cognitive decline, lower baseline cognition may account for racialized individuals disproportionately developing dementia (Weuve et al., 2018).

In this analysis, well-established social determinants of cognitive health such as income, social standing, social support, and having a hobby were not associated with changes in executive function in our sample. This is in contrast to other research that has found longitudinal associations between social factors and cognitive aging (Seeman et al., 2001), as well as cross-sectional associations between social determinants (i.e., income, social standing, having a hobby, social support) and executive function (Stinchcombe and Hammond, 2021). Surprisingly, education was negatively associated with change in executive function in our sample. Based on the cognitive reserve theory, and previous work looking at social determinants of cognition, it was predicted that higher educational attainment would act as a protective factor for executive functioning (Stern, 2012; Majoka and Schimming, 2021). Additionally, cross-sectional findings using data from the CLSA found a positive association between education and executive function such that participants with higher education had better executive function scores at baseline (Stinchcombe and Hammond, 2021). Much of the cognitive reserve literature has focused on education obtained in early-life as protective against dementia (EClipSE Collaborative Members et al., 2010), and the measure of education in this study does not specify when the education was obtained. Moreover, other work suggests that education is predictive of baseline cognition, but not rate of cognitive change (Wilson et al., 2019). Future longitudinal work should further clarify the role of education and changes in executive function.

A strength of this study includes a large sample size of almost 30,000 Canadian mid-age and older adults. Additionally, due to the breadth of variables in the CLSA dataset we were able to include many health factors as well as social determinants of health in our model. This allowed us to better understand the extent that social determinants are associated with executive function over time, while controlling for other health factors that may impact executive function. In addition, we used an objective measure of executive function collected at two time points and used a statistical method (reliable change index; RCI) to control for measurement error. The robustness of our findings was confirmed when using a simple discrepancy score in our sensitivity analysis. While it is interesting to compare the two models, it should be noted that the RCI provides a more reliable estimate of changes in cognition in comparison to the discrepancy score.

With respect to limitations, a 3-year follow-up time frame, as well as being limited to two data time points, reduces the ability to explore trajectories of change and best understand the correlates of changes in executive function over time. Additional longitudinal data is needed to better understand the long-term impact of social determinants on cognitive aging. While the MAT is an objective measure of executive function suitable for population health studies focused on aging, it is not a detailed and multi-faceted assessment of cognitive functions. This study was also limited by covariates not captured by the dataset. In particular, we were precluded from directly examining stress experiences known to impact cognitive aging and instead relied on minority identities more likely to experience stress (e.g., sexual orientation). Future research looking at trajectories of executive function should include measures of stress and examine intersecting social identities. The covariates in our study were based on self-report which may underestimate the true prevalence of some health conditions and behaviors (e.g., alcohol consumption, heart disease, and stroke). Finally, the sample was generally healthy and well-resourced with only a small number of minoritized individuals, reducing statistical power and limiting generalizability of the results. Data collection initiatives seeking to understand cognitive aging should engage with community groups to ensure visibility of and representation from members of minoritized groups.

A growing body of evidence highlights the role of social determinants and, in particular, social identities, as risk factors for cognitive aging. Research points to minority stress experiences accumulated across the lifespan contributing to brain health (Forrester et al., 2019). In our analysis, minority identities were not statistically associated with changes in executive function over a 3-year period, after accounting for other covariates. Future research should investigate cognitive aging trajectories and dementia risk over longer periods of time and consider engaging with members of minoritized groups and oversampling participants with minority identities.

## Data availability statement

The data analyzed in this study is subject to the following licenses/restrictions: Data are available to approved data users. Requests to access these datasets should be directed to https://www.clsa-elcv.ca/data-access.

## Ethics statement

The current analyses were reviewed and approved by University of Ottawa’s Research Ethics Board (REB). The participants provided their written informed consent to participate in this study. The CLSA was reviewed and approved by multiple Research Ethics Boards (REB) and all participants gave informed consent to participate.

## Author contributions

AS and NH contributed to the conception and design of the study. NH completed the main analyses. AS and SH contributed to the writing of the manuscript. AS, NH, and SH edited the draft. AS supervised the work and secured funding. All authors contributed to the article and approved the submitted version.

## Funding

This work was supported by a grant from the Alzheimer’s Society of Canada Research Program awarded to AS. NH is funded by the Frederick Banting and Charles Best Canada Graduate Scholarship Doctoral Awards (CGS-D) program. SH is funded by a SSHRC Canada Graduate Scholarship Doctoral Award (CGS-D).

## Conflict of interest

The authors declare that the research was conducted in the absence of any commercial or financial relationships that could be construed as a potential conflict of interest.

## Publisher’s note

All claims expressed in this article are solely those of the authors and do not necessarily represent those of their affiliated organizations, or those of the publisher, the editors and the reviewers. Any product that may be evaluated in this article, or claim that may be made by its manufacturer, is not guaranteed or endorsed by the publisher.
